# Pharmacodynamic Evaluation of the Gexia Zhuyu Decoction in the Treatment of NAFLD and the Molecular Mechanism Underlying the TRPM4 Pathway Regulation

**DOI:** 10.1155/2021/3364579

**Published:** 2021-11-30

**Authors:** Yao-Wei Zhao, Jing Yang, Jie Niu, Tong Wang, Xin-Dan Liang, Yang Ren, Rui Wang

**Affiliations:** ^1^College of Basic Medical Science, Heilongjiang University of Chinese Medicine, Harbin, Heilongjiang 150040, China; ^2^College of Pharmacy, Heilongjiang University of Chinese Medicine, Harbin, Heilongjiang 150040, China

## Abstract

Nonalcoholic fatty liver disease (NAFLD) is a clinicopathological syndrome of abnormal lipid deposition in the liver mediated by nonalcohol intake. The Gexia Zhuyu decoction, a classic traditional Chinese medicine compound, is widely used in the clinical treatment of NAFLD. However, its specific efficacy and underlying mechanisms have not been elucidated yet. This study aimed to quantitatively evaluate the efficacy of the Gexia Zhuyu decoction using pharmacodynamics and to explore its molecular mechanisms in conjunction with proteomics. High-fat diets and methionine choline-deficient diets were used to induce various NAFLD progression stages in mouse models. The effects of oral Gexia Zhuyu decoction administration on NAFLD were evaluated by measuring the serum and liver indicators of the treated mice before and after drug intervention and by comparing the changes in liver tissue. Liver TRPM4 mRNA and protein levels were measured using reverse transcription-polymerase chain reaction and Western blotting, respectively. Experimental data showed that serum ALT, AST, and liver triglyceride (TG) levels in each disease stage group of drug intervention mice decreased, and high-density lipoprotein (HDL) and superoxide dismutase (SOD) levels increased. Liver TG levels decreased after drug intervention in the liver fibrosis mice, but serum TG levels increased. Furthermore, cellular fatty changes, inflammatory changes, and fibrous tissue proliferation were all relieved. The TRPM4 protein and mRNA levels in the liver tissue were decreased, and the microRNA (miRNA)-24 expression was increased. The Gexia Zhuyu decoction has a clear therapeutic effect at each stage of NAFLD. It likely acts by altering miRNA-24 expression and regulating the target TRPM4 protein pathway to achieve NAFLD treatment.

## 1. Introduction

Nonalcoholic fatty liver disease (NAFLD), commonly associated with chronic liver damage, is usually accompanied by metabolic complications, such as elevated blood lipid and blood glucose levels, uncontrolled blood pressure, and vascular dysfunction. NAFLD is progressive and includes three main pathological stages: nonalcoholic simple fatty liver (NAFL), nonalcoholic steatohepatitis (NASH), and progressive liver fibrosis [1]. Due to lifestyle and dietary changes, the global incidence of the disease has increased exponentially in recent years, and it has become a high-risk disease that infringe on the normal physiological functioning of the liver [2]. Epidemiological surveys [3, 4] have shown that the prevalence of NAFLD in China has risen sharply in the past ten years, by as much as 26–45%, and is now the most common chronic liver disease in the country. There are many pathogenic factors associated with NAFLD, and its pathogenesis is not yet clear. At present, the most widely accepted theory is the “two-hit hypothesis” [5]. Therefore, symptomatic treatment is often employed clinically, including lipid-lowering and glucose and lipid metabolism improvements. However, most lipid-lowering drugs degrade blood lipid levels and promote lipid entry into the liver for metabolism and excretion, which can easily lead to liver damage and liver fat deposition [6]. Therefore, it has become an urgent task to find a drug that has clear therapeutic effects, is safe, and has minimal side effects.

The Gexia Zhuyu decoction, a representative compound of traditional Chinese medicine used to promote blood circulation and alleviate blood stasis, is widely used in the clinical treatment of various chronic liver diseases. Studies have shown that Chinese medicines with these effects can improve peripheral vascular microcirculation, correct abnormal connective tissue metabolism, strengthen the body's ability to transport oxygen and nutrients to the liver, reverse tissue ischemia and hypoxia, and help the liver repair itself. In traditional Chinese medicine, it is believed that the main causes of NAFLD are irregular eating habits and overeating, which lead to an increased liver load, and the lack of adequate physical exercise. Prolonged imbalance between work and rest leads to further liver damage and induces NAFLD [7]. Studies have shown that the chemical components of the Gexia Zhuyu decoction have good protective effects against liver damage at each pathological stage of NAFLD. The active components of this single medicine have clear lipid-lowering, anti-inflammatory, and antioxidant properties. For example, the single Chinese medicine hydroxysafflor yellow A has antifibrosis, analgesic, and antioxidant properties. It promotes blood circulation, alleviates blood stasis, and is believed to have antitumor effects [8–13]. Furthermore, it protects liver tissues from oxidative stress damage, reduces the degree of liver cell edema, and promotes liver tissue repair. Paeoniflorin has a therapeutic effect on carbon tetrachloride-induced chronic liver injury and fibrosis in rats, while ferulic acid has antioxidant effects, can lower blood lipids, and reduce blood cholesterol levels [14–17]. In addition, traditional Chinese medicines are mostly sourced from animals and plants. Thus, compared with those of the chemical extracts or synthetic agents used in Western medicine, the medicinal effects of traditional Chinese medicines are mild, with minimal side effects. The Gexia Zhuyu decoction is prepared as an arrangement of individual natural medicinal materials in combination, chosen based on the patient's condition, to adapt to the law of occurrence and disease development. Furthermore, it regulates abnormal physiological processes in the body and restores homeostasis by making use of the multilevel, multitarget, and multipathway nature of Chinese herbal compound preparations.

Transient receptor potential channel M (TRPM) is a family of cation channels located on the mammalian cell membrane. They mediate the polarization of cations in the cell membrane, regulate cell growth, metabolism, and intracellular material exchange, and maintain the intracellular environment. Stability plays an important role in this process [18]. Studies have found that when the ATP content decreases, the opening of the TRPM4 channel causes the sodium ion pump to malfunction, resulting in an increase in intracellular permeability, which, in turn, induces cell swelling, rupture, and death [19]. Therefore, TRPM4 plays an important role in cell necrosis and apoptosis [20, 21]. Studies have shown that chronic liver disease is closely related to the abnormal expression of microRNAs (miRNAs) [22]; using related databases to predict the target of TRPM4 revealed that miRNA-24 and TRPM4 had a strong association, suggesting that TRPM4 is very likely to be a target gene of miRNA-24. Experiments have shown that miRNA-24 can affect many signaling pathways by regulating multiple target genes, thereby changing the process of cell proliferation, migration, and apoptosis [23]. Preliminary experiments showed that TRPM4 is very important in the progression of NAFLD, though there are no existing reports on miRNAs regulating TRPM4. Therefore, in-depth exploration of the regulatory relationship between TRPM4 and miRNA-24 is of high research value.

We selected high-fat diets (HFD) and methionine choline-deficient (MCD) diets to reproduce the three main pathological stages in NAFLD progression, namely, NAFL, NASH, and nonalcoholic fatty liver fibrosis in this study. First, pharmacodynamic analysis of the effect of the Gexia Zhuyu decoction at each pathological stage of NAFLD was performed based on the changes in serum, biochemical indicators, and pathological tissues. Furthermore, we observed the effect of the Gexia Zhuyu decoction on TRPM4 expression and used the biological information database to predict the TRPM4 target. Finally, we verified the relationship between TRPM4 and miRNA using PCR target regulation relationship and proffered the mechanism by which traditional Chinese medicine promotes blood circulation and alleviates blood stasis to prevent and treat diseases such as NAFLD.

## 2. Materials and Methods

### 2.1. Preparation of Gexia Zhuyu Decoction

The Gexia Zhuyu decoction consists of the following components: *Prunus persica* L. (grinding mud), 9 g, dried faeces of *Trogopterus xanthipes* Milne-Edwards (fried), 6 g, *Paeonia obovata*, 6 g, *Paeonia suffruticosa*, 6 g, *Angelica sinensis* (Oliv.) Diels, 9 g, *Corydalis ternata* (Nakai) Nakai, 3 g, *Ligusticum chuanxiong* S, 6 g, *Lindera aggregata* (Sims) Kosterm., 6 g, *Glycyrrhiza uralensis* Fisch., 9 g, *Carthamus tinctorius* L., 9 g, *Cyperus rotundus* L., 4.5 g, and *Poncirus trifoliata* L., 4.5 g. These medicinal materials were purchased from the Harbin Tongrentang Pharmacy (Harbin, China). The research team optimized the soaking time, decoction duration, water dosage, and other factors of the medicinal materials through single factor investigations and orthogonal experiments at an earlier stage and obtained the best preparation methodology for the Gexia Zhuyu decoction. Water was added ten times the compound dose, and the mixture was fully soaked for 60 min and decocted twice for 2 h each. The Gexia Zhuyu decoction was prepared as a mixture of these two decocted solutions. Through the HPLC analysis of Gexia Zhuyu decoction, the determination results showed that the total amount of hydroxyl safflower yellow pigment A in Gexia Zhuyu decoction was greater than 0.25 mg/mL and the total amount of ferulic acid was greater than 0.22 mg/mL, in line with the standard (concentration of hydroxyl safflower yellow pigment A standard: 0.105 mg/mL ferulic acid standard: 0.0636 mg/mL), can be used for intragastric administration, into the pharmacodynamics study.

### 2.2. Laboratory Animals and Feed

Six-to-eight-week-old male C57BL/6J mice (Experimental Animal Center of Heilongjiang University of Traditional Chinese Medicine, Harbin, China) weighing 20 ± 2 g were used (certificate number: SCXK (HEI) 2016-0001). The experimental animal room was well ventilated, with a 12 h/12 h light and dark cycle, a room temperature of 22–25°C, and relative humidity of 45–60% [24]. Ordinary feed was purchased from the Experimental Animal Center of Heilongjiang University of Traditional Chinese Medicine (Harbin, China); 45% kcal high-fat diet-induced obesity (DIO) diets were purchased from Research Diets Inc. (New Brunswick, NJ, USA); and MCD diets were purchased from Moldiets (Beijing, China) [25, 26]. The study was performed in accordance with the ARRIVE guidelines. Ethical approval was obtained from the Heilongjiang University of Chinese Medicine Laboratory Animal Management and Use Committee, no. 2019031101.

### 2.3. Instruments

Low-temperature high-speed centrifuge (MIKRO 220, Schwartzwald, Germany), paraffin slicer (Leica, Frankfurt, Germany), slicer (Leica), optical microscope (Leica), vertical electrophoresis tank (Bio-Rad, Hercules, CA, USA), transfer electrophoresis tank (Bio-Rad), and fluorescence quantitative PCR instrument (Applied Biosystems, Foster City, CA, USA) were used for the experiments.

### 2.4. Reagent

The PCIII ELISA kit (Shanghai Huzheng Biotechnology Co., Ltd., Shanghai, China), IV-C and LN ELISA kits (Nanjing SenBeiJia Biological Technology Co., Ltd., Nanjing, China), HA and AST ELISA kits (Shanghai Jianglai Biotechnology Co., Ltd., Shanghai, China), ALT, TG, and TC ELISA kits (Shanghai Enzyme Biotechnology Co., Ltd., Shanghai, China), SYBR Green PCR Master Mix kit (Applied Biology, Irvine, CA, USA), Fermentas K1622 RT reverse transcription kit (Massachusetts Biomedical Initiatives, Worcester, MA, USA), absolute ethanol (Sinopharm Chemical Reagent Co., Ltd., Shanghai, China), chloroform (Shanghai Shenggong Co., Ltd., Bioengineering, Shanghai, China), isopropanol (Sinopharm Group Chemical Reagent Co.), TRIzol (Invitrogen, Carlsbad, CA, USA), SDS-PAGE gel compounding reagent box (Biyuntian Biotechnology Co., Ltd., Shanghai, China), rabbit antihepatitis B virus pre-X protein antibody, and goat anti-rabbit IgG H and L (HRP) (Abcam, Cambridge, UK) were used in this study.

### 2.5. Experimental Design

After environmental adaptation for 1 week, 120 C57BL/6J mice were grouped according to the three main pathological stages of NAFLD: the NAFL group, NASH group, and nonalcoholic fatty liver fibrosis group. Each group consisted of 40 mice, and the animal models were used to simulate NAFLD disease.

Among them, the three pathological stages are divided into specific groups. The NAFL group also includes the following: NAFL blank control group, which consisted of healthy mice as the research control (control group, NAFL); the NAFL blank healthy mice gavage Gexia Zhuyu decoction group to verify the diaphragm side effects of Gexia Zhuyu decoction (CG-NAFL + GXZY); the NAFL model group, in which the mice induced with feed to establish the NAFL model (model group, NAFL); and the NAFL Gexia Zhuyu decoction group, in which mice successfully modeled NAFL mice by gavage with diaphragm and Gexia Zhuyu decoction for pharmacodynamic research and mechanism research (GXZY-NAFL). Each group consisted of 10 mice.

The NASH group included the following: NASH blank control group, which consisted of healthy mice as research controls (control group, NASH); the NASH blank gavage Gexia Zhuyu decoction group to verify the side effects of Gexia Zhuyu decoction (CG-NASH + GXZY); the NASH model group, in which the mice were induced by feed (model group, NASH); and the NASH Gexia Zhuyu decoction group, in which the mice successfully modeled NASH mice by gavage with diaphragm and Gexia Zhuyu decoction for pharmacodynamic research and mechanism research (GXZY-NASH). Each group consisted of 10 mice.

The nonalcoholic fatty liver fibrosis group included the following: the nonalcoholic fatty liver fibrosis blank control group was to set up healthy mice as the research control at this pathological stage (control group, fibrosis); the nonalcoholic fatty liver fibrosis blank control Gexia Zhuyu decoction group was used to verify the effect of Gexia Zhuyu decoction on healthy mice (CG-fibrosis + GXZY); the nonalcoholic fatty liver fibrosis group model group feed-induced establishment of the nonalcoholic fatty liver fibrosis model (model group, fibrosis); and the nonalcoholic fatty liver fibrosis Gexia Zhuyu decoction group consisted of mice that successfully modeled fibrotic mice with Gexia Zhuyu decoction for pharmacodynamic and mechanism research (GXZY-fibrosis). Each group consisted of 10 mice.

### 2.6. Serum and Biochemical Indicators

ELISA was used to determine serum aspartate aminotransferase (AST) and alanine aminotransferase (ALT) levels and liver triglyceride (TG) levels in the three mice groups. The typical indicators of each group were further measured. Serum total cholesterol (TC), high-density lipoprotein (HDL), low-density lipoprotein (LDL), and liver TC content were measured in the NAFL group mice to assess the improvement in the lipid metabolism disorders. Serum TC, tumor necrosis factor (TNF)-*α*, interleukin (IL)-6, liver TC, malondialdehyde (MDA), and superoxide dismutase (SOD) levels were measured in the NASH group mice to evaluate the anti-inflammatory and antioxidant capacity of the Gexia Zhuyu decoction [27]. Serum procollagen type III (PCIII), type IV collagen (IV-C), laminin (LN), hyaluronic acid (HA), and liver MDA and SOD levels were measured in the liver fibrosis group mice to evaluate the effect of the Gexia Zhuyu decoction on liver fibrosis [28].

### 2.7. Histopathological Examination

The liver tissue samples were fixed with 4% formaldehyde, dehydrated, made transparent, and then waxed and embedded to obtain tissue wax blocks. The wax blocks were cut into slices 3–5 *μ*m thick, and hematoxylin and eosin (HE) and Masson's staining were performed [24]. The changes in tissue morphology before and after drug administration were observed under a microscope.

### 2.8. Western Blotting

A small liver tissue sample was obtained and cleaned to remove blood. The liver tissue was transferred to a homogenizer, and a lysate mixture (RIPA : PMSF = 100 : 1) was added. The tissue was ground in a liquid nitrogen environment and centrifuged at 12,000 rpm for 5 min at 4°C. The liver tissue protein was extracted, and protein concentration was measured using the BCA protein assay kit. The proteins were then electrophoresed on a 10% SDS-PAGE gel, transferred to a PVDF membrane, and onto a blocking solution at 4°C for 2 h. Subsequently, the proteins were incubated with the PVDF membrane and the primary antibody (rabbit antihepatitis B virus pre-X protein antibody) overnight in a refrigerator at 4°C. The membrane was then washed five times with TBST (Tris-buffered saline with 0.1% Tween® 20 detergent) for 10 min and reacted with the secondary antibody (goat anti-Rabbit IgG H and L (HRP)) at room temperature for 1 h. The resulting solution was mixed in the ECL kit; chromogenic solutions A and B were mixed in a 1 : 1 ratio according to the manufacturer's instructions and dropped on the PVDF film for development [29]. The gel chemiluminescence analysis software was used for quantitative analysis according to the brightness of the protein bands.

### 2.9. RT-PCR Analysis

Total RNA was extracted from a small amount of frozen liver tissue using TRIzol™ reagent after grinding, and 2 *μ*L of loading buffer was added to 4 *μ*L of RNA sample from each group and mixed properly. A 1% agarose gel was prepared for electrophoresis to verify RNA band completeness. cDNA was transcribed using the kit, and the target fragment was amplified and synthesized using DNA polymerase. The data were analyzed using the 2^−ΔΔCT^ method, and *β*-actin was used as the internal reference gene to record the relative quantitative value of the target gene expression in each group for statistical analysis [30]. Specific primer information is given in Table 1.

### 2.10. Regulatory Relationship between miRNA-24 and TRPM4

Using the TargetScan [31, 32] and microRNA.org [33] databases for bioinformatics target prediction, we determined that TRPM4 was the target protein of miRNA-24. miRNA-24 expression in the liver tissue was detected by RT-PCR using U6 as the internal reference gene. Combining the aforementioned analysis results of Western blotting (WB) and RT-PCR for TRPM4, the regulatory relationship between miRNA-24 and the TRPM4 pathway was clarified, and the molecular mechanism of action of the Gexia Zhuyu decoction in the treatment of NAFLD was further revealed.

### 2.11. Data Analysis

The SPSS 13.0 software (SPSS Inc., Chicago, IL, USA) was used to process the data, and all results were obtained as the mean ± standard deviation (SD); the difference between each group was calculated by analysis of variance. *P* < 0.05 indicated statistical significance.

## 3. Results

### 3.1. Efficacy Evaluation

All mice were alive at the end of the experiment. CG-NAFL, CG-NASH and CG-fibrosis mice in the three disease groups were generally in good condition and full of energy, with no abnormality in food intake, appetite, flexibility, and activity. Their furs felt smooth and were oily in appearance, and their body mass showed a linear increase during the later stage of the experiment. Sensitivity to external stimuli and urine and fecal volume in CG-NAFL, CG-NASH, and CG-fibrosis mice were also normal. MG-NAFL and MG-NASH mice showed dry hair, slow movement, rapid weight gain, multicolored urine, small and thin stools, docile temperament, and no irritability. Mice in MG-fibrosis grew gradually lethargic showing slow movement, decreased food intake and appetite, dull and disorderly hair, lack of luster, and severe hair loss. Individual mice also presented with epistaxis, weight loss, slow response to external stimuli, and decreased stool and urine output. There were no obvious changes in the mental states of CG-NAFL + GXZY, CG-NASH + GXZY and CG-fibrosis + GXZY mice in the three disease groups. The mice in this group had smooth hair and were flexible and active, with stable food intake and relatively normal indicators. The coats of GXZY-NAFL, GXZY-NASH and GXZY-fibrosis mice in each disease group were slightly shiny, and they showed reduced daily dietary intake, acceptable activity level, and better mental state and slower weight loss than MG-NAFL, MG-NASH, and MG-fibrosis mice.

### 3.2. Serum and Biochemical Index Detection

As given in Table 2, the levels of ALT, AST, and liver and serum TG at three pathological stages of CG-NAFL, CG-NASH, and CG-fibrosis and CG-NAFL + GXZY, CG-NASH + GXZY, and CG-fibrosis + GXZY mice did not change significantly, suggesting that the Gexia Zhuyu decoction had almost no toxic effect on the body, while also eliminating the interference of drugs with the measured indicators. NAFL serum biochemical analysis results showed that the serum TG, AST, and ALT levels of the MG-NAFL mice were increased, the serum TG and liver tissue TG levels of the GXZY-NAFL mice were decreased, and the serum ALT and AST levels were decreased. After administration of the decoction to the NASH group, serum TG, AST, and ALT levels and liver tissue TG levels of the CG-NASH and CG-NASH + GXZY mice did not change significantly. Compared with those of the CG-NASH mice, the serum TG, AST, and ALT levels and liver TG levels of the MG-NASH mice were increased. In GXZY-NASH mice, serum TG, AST, and ALT levels and liver TG levels decreased. In the liver fibrosis group, serum ALT and AST levels of CG-fibrosis and CG-fibrosis + GXZY mice did not change significantly after the last dose administration. In MG-fibrosis mice, serum ALT and AST levels increased, serum TG levels decreased significantly, and liver TG levels increased, suggesting a lipid metabolism disorder. Furthermore, fatty deposits were observed in the MG-fibrosis mice, indicating that the liver was severely damaged and liver function was impaired. Compared with those of MG-fibrosis mice, the serum ALT and AST levels of GXZY-fibrosis group mice were decreased to varying degrees. Serum index TG levels rebounded, and liver TG levels decreased. Therefore, after the oral intervention of Gexia Zhuyu decoction, liver fat accumulation improved, lipid metabolism disorder was corrected, and excess lipids were metabolized through blood via the circulatory system, resulting in increased blood lipids and increased serum TG.

### 3.3. Specific Indicator Detection

#### 3.3.1. NAFL Group Index Measurement

**Figure 1:**
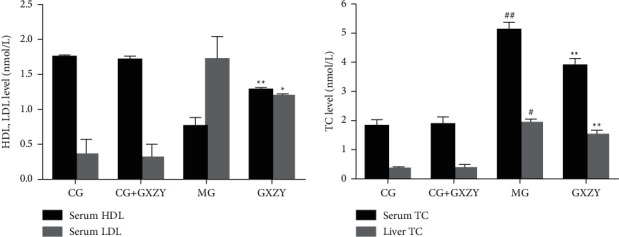
Changes in (a) HDL and LDL levels, and (b) TC levels after Gexia Zhuyu decoction administration. Compared with the control group (CG-NAFL, 1.75 ± 0.02 nmol/L, 0.35 ± 0.22 nmol/L, 2.03 ± 0.13 nmol/L, and 0.35 ± 0.06 nmol/L, *n* = 10), ^##^*P* < 0.01, ^#^*P* < 0.05; compared with the model group (MG-NAFL, 0.76 ± 0.12 nmol/L, 1.72 ± 0.32 nmol/L, 4.85 ± 0.14 nmol/L, and 1.18 ± 0.43 nmol/L, *n* = 10), ^∗∗^*P* < 0.01, ^∗^*P* < 0.05. HDL, high-density lipoprotein; LDL, low-density lipoprotein; TC, total cholesterol.

As shown in Figure 1, after 8 weeks of Gexia Zhuyu decoction administration, serum HDL, LDL, and liver TC levels in CG-NAFL (1.75 ± 0.02 nmol/L, 0.35 ± 0.22 nmol/L, 0.35 ± 0.06 nmol/L, *n* = 10) and CG-NAFL + GXZY(1.71 ± 0.05 nmol/L, 0.31 ± 0.19 nmol/L, 0.37 ± 0.08 nmol/L, *n* = 10) mice showed no significant changes. Whereas, the LDL levels (1.19 ± 0.03 nmol/L, *n* = 10) and serum and liver TC levels (3.53 ± 0.35 nmol/L, 0.86 ± 0.42 nmol/L, *n* = 10) decreased, and serum HDL levels (1.28 ± 0.03 nmol/L, *n* = 10) increased in GXZY-NAFL mice.

#### 3.3.2. NASH Group Indicators

**Figure 2:**
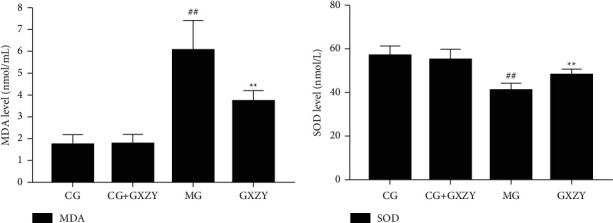
Changes in (a) MDA and (b) SOD levels in the NASH group after the administration of the Gexia Zhuyu decoction. Compared with the control group (CG-NASH, 1.71 ± 0.46 nmol/ml and 57.18 ± 4.20 nmol/mL, *n* = 10), ^##^*P* < 0.01, ^#^*P* < 0.05; compared with the model group (MG-NASH, 6.06 ± 1.37 nmol/ml and 41.13 ± 3.11 nmol/ml, *n* = 10), ^∗∗^*P* < 0.01, ^∗^*P* < 0.05. MDA, malondialdehyde; SOD, superoxide dismutase.

**Figure 3:**
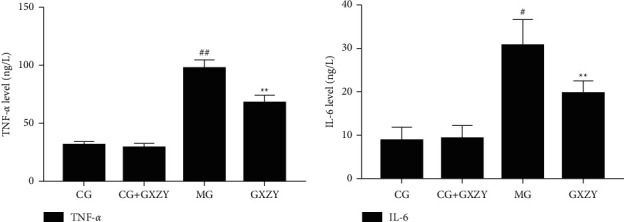
Changes in (a) TNF-*α* and (b) IL-6 levels after Gexia Zhuyu decoction administration. Compared with the control group (CG-NASH, 31.35 ± 3.10 ng/L, and 8.81 ± 3.00 ng/L, *n* = 10), ^##^*P* < 0.01, ^#^*P* < 0.05; compared with the model group (MG-NASH, 97.71 ± 7.00 ng/L 30.66 ± 6.00 ng/L, *n* = 10), ^∗∗^*P* < 0.01. IL-6, interleukin-6; TNF-*α*, tumor necrosis factor-*α*.

**Figure 4:**
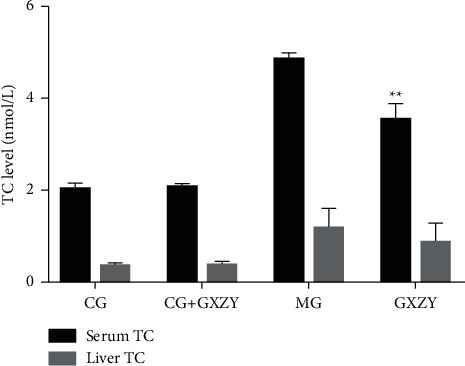
Changes in (A) serum TC and (B) liver TC levels after the Gexia Zhuyu decoction administration. Compared with the model group (MG-NASH, 5.12 ± 0.25 nmol/L and 1.92 ± 0.14 nmol/L, *n* = 10), ^∗∗^*P* < 0.01. TC, total cholesterol.

As shown in Figures 2–4, after 8 weeks of decoction administration, there were no significant changes in serum TC, TNF-*α*, and IL-6 levels and liver MDA, SOD, and TC levels in CG-NASH (1.82 ± 0.21 nmol/L, 31.35 ± 3.10 ng/L, 8.81 ± 3.00 ng/L, 1.71 ± 0.46 nmol/ml, 57.18 ± 4.20 nmol/ml, and 0.35 ± 0.06 nmol/L, *n* = 10) and CG-NASH + GXZY (1.88 ± 0.25 nmol/L, 29.26 ± 3.30 ng/L, 9.21 ± 3.00 ng/L, 1.78 ± 0.43 nmol/ml, 55.36 ± 4.50 nmol/mL, and 0.37 ± 0.08 nmol/L, *n* = 10) mice. In MG-NASH mice, serum TC, TNF-*α*, and IL-6 levels and liver MDA and TC levels (5.12 ± 0.25 nmol/L, 97.71 ± 7.00 ng/L, 30.66 ± 6.00 ng/L, 6.06 ± 1.37 nmol/mL, and 1.92 ± 0.14 nmol/L, *n* = 10) increased, while the SOD content (41.13 ± 3.11 nmol/mL) decreased. Compared with MG-NASH mice, in GXZY-NASH mice, serum TC, TNF-*α*, and IL-6 levels and liver MDA and TC contents (3.89 ± 0.24 nmol/L, 67.93 ± 6.10 ng/L, 19.70 ± 2.85 ng/L, 3.73 ± 0.47 nmol/ml, and 1.51 ± 0.16 nmol/L, *n* = 10) decreased after decoction administration, and the SOD content (48.11 ± 2.71 nmol/ml) increased.

#### 3.3.3. Liver Fibrosis Group

**Figure 5:**
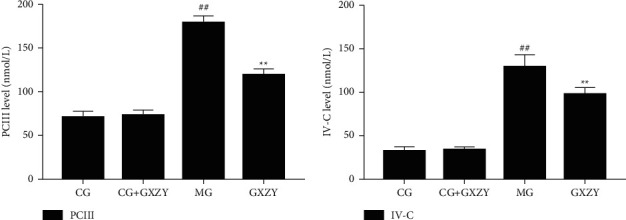
Changes in (a) PCIII and (b) IV-C levels in mice after the Gexia Zhuyu decoction administration. Compared with the control group (CG-fibrosis, 70.63 ± 6.82 nmol/L and 32.35 ± 4.78 nmol/L, *n* = 10), ^##^*P* < 0.01; compared with the model group (MG-fibrosis, 178.51 ± 7.59 nmol/L and 129.57 ± 13.75 nmol/L, *n* = 10), ^∗∗^*P* < 0.01. PCIII, procollagen type III; IV-C, type IV collagen.

**Figure 6:**
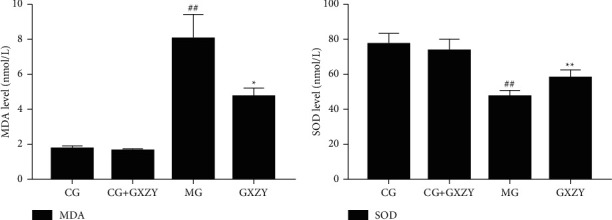
Changes in (a) MDA and (b) SOD levels in the fibrosis group after the administration of the Gexia Zhuyu decoction. Compared with the control group (CG-fibrosis, 1.75 ± 0.16 nmol/L and 77.36 ± 6.15 nmol/L, *n* = 10), ^##^*P* < 0.01; compared with the model group (MG-fibrosis, 8.06 ± 1.37 nmol/L and 47.27 ± 3.71 nmol/L, *n* = 10), ^∗∗^*P* < 0.01, ^∗^*P* < 0.05. MDA, malondialdehyde; SOD, superoxide dismutase.

**Figure 7:**
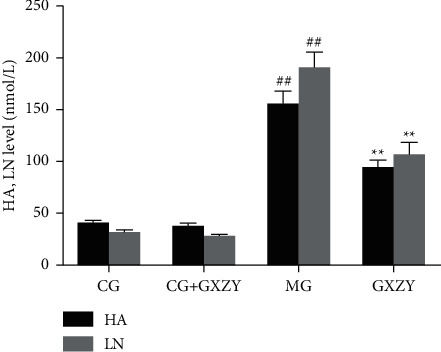
Changes in (A) HA and (B) LN levels after the Gexia Zhuyu decoction administration. Compared with the control group (CG-fibrosis, 39.63 ± 3.31 nmol/L and 30.35 ± 3.21 nmol/L, *n* = 10), ^##^*P* < 0.01; compared with the model group (MG-fibrosis, 154.51 ± 13.09 nmol/L and 189.57 ± 15.75 nmol/L, *n* = 10), ^∗∗^*P* < 0.01. HA, hyaluronic acid; LN, laminin.

As shown in Figures 5–7, after 8 weeks of drug intervention, there was no significant change in serum PCIII, IV-C, LN, and HA, MDA, SOD levels in the CG-fibrosis (70.63 ± 6.82 nmol/L, 32.35 ± 4.78 nmol/L, 30.35 ± 3.21 nmol/L, 39.63 ± 3.31 nmol/L, 1.75 ± 0.16 nmol/L, and 77.36 ± 6.15 nmol/L, *n* = 10) and CG-fibrosis + GXZY mice (72.85 ± 6.02 nmol/L, 33.78 ± 3.25 nmol/L, 27.38 ± 2.25 nmol/L, 37.05 ± 3.52 nmol/L, 1.62 ± 0.13 nmol/L, and 73.56 ± 6.50 nmol/L, *n* = 10) mice, suggesting that the components of the Gexia Zhuyu decoction did not affect the four indicators of liver fibrosis in the healthy liver. Thus, administration of the decoction produced no side effects. Whereas, the serum PCIII, IV-C, LN, and HA, MDA levels of the MG-fibrosis mice increased significantly (178.51 ± 7.59 nmol/L, 129.57 ± 13.75 nmol/L, 189.57 ± 15.75 nmol/L, 154.51 ± 13.09 nmol/L, and 8.06 ± 1.37 nmol/L, *n* = 10), indicating that the MG-fibrosis mice developed liver fibrosis, and those of the GXZY-fibrosis mice all decreased to varying degrees (118.53 ± 7.62 nmol/L, 73.51 ± 7.79 nmol/L, 105.51 ± 12.79 nmol/L, 93.53 ± 8.12 nmol/L, and 4.73 ± 0.47 nmol/L, *n* = 10).

### 3.4. Pathological Examination

**Figure 8:**
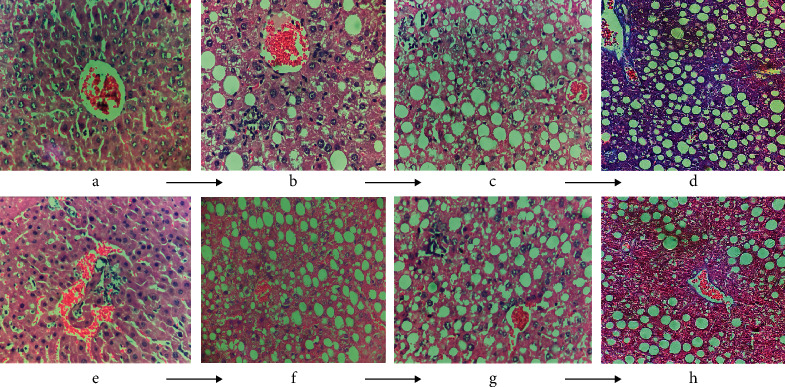
The effect of the Gexia Zhuyu decoction administration on the pathological morphology of the liver in NAFLD mice. (d, h) Stained with Masson's stain, and the remaining sections were stained with H&E and observed under a 400× microscope. (a, e) Control group. (b, f) NAFL model group and Gexia Zhuyu decoction treatment group, respectively. (c, g) HASH model group and Gexia Zhuyu decoction treatment group, respectively. (d, h) NAFL model group and Gexia Zhuyu decoction treatment group, respectively.

As shown in Figure 8, after 8 weeks of decoction administration, the tissue sections of the GXZY-NAFL, GXZY-NASH, and GXZY-fibrosis mice at each pathological stage, compared with the corresponding tissue sections of the MG-NAFL, MG-NASH, and MG-fibrosis mice, showed slightly reduced fatty vacuoles, and the structures of a small number of hepatocytes showed a trend of improvement. The liver tissue sections of the CG-NAFL, CG-NASH, and CG-fibrosis mice were clear and complete, with normal liver lobule structures. The liver cells were arranged in strips, radially distributed around the central vein, and showed no steatosis. The MG-NAFL mice showed fatty lesions in the liver lobules. The structure of the liver lobules in the MG-NASH mice was damaged, and the liver cells showed bullous steatosis. Inflammatory cell infiltration was seen in the lobules and portal area. Liver cells in the MG-fibrosis mice showed fatty degeneration and enlargement; they were mainly vesicular, showing cell necrosis and infiltration of inflammatory factors. The dark blue color indicates fibrous hyperplasia in the portal area and surrounding sinuses. After drug intervention, the fatty vacuoles were slightly reduced, and the structures of a number of liver cells improved. After drug intervention in the GXZY-fibrosis, the blue-stained area was reduced, and fibrosis was inhibited. Evidently, the Gexia Zhuyu decoction regulated liver cell steatosis, inflammatory changes, fibrosis, and hyperplasia, thereby protecting the liver from damage and showing treatment potential for each stage of NAFLD.

### 3.5. Western Blotting

**Figure 9:**
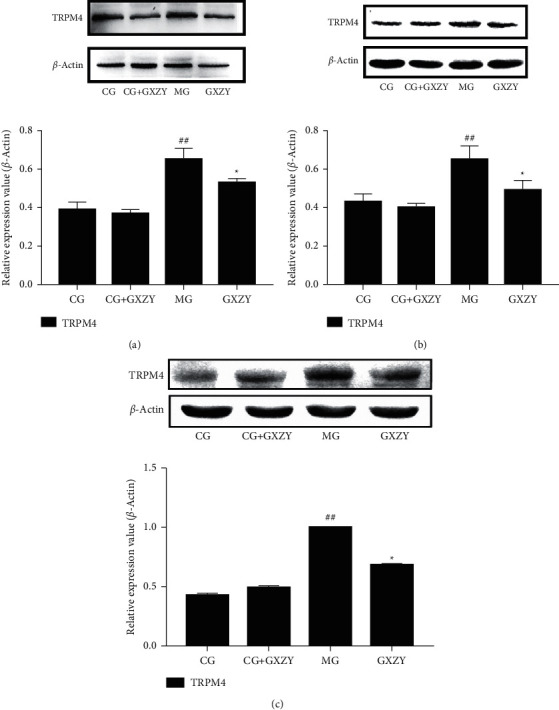
Expression bands and relative expression levels of the TRPM4 protein at the three pathological stages of NAFLD. (a) NAFL group. (b) NASH group. (c) Nonalcoholic fatty liver fibrosis group. Compared with the control group, ^##^*P* < 0.01; compared with the model group, ^∗^*P* < 0.05.

The protein and internal control expression bands are shown in Figure 9. There was no significant difference in color depth and width between the CG-NAFL, CG-NASH, and CG-fibrosis and CG-NAFL + GXZY, CG-NASH + GXZY, and CG-fibrosis + GXZY mice proteins at the three pathological stages of NAFLD. Compared with those of the CG-NAFL, CG-NASH, and CG-fibrosis mice proteins, the MG-NAFL, MG-NASH, and MG-fibrosis mice protein bands were darker and thicker, suggesting that TRPM4 protein expression was abnormally increased in NAFLD. Compared with the MG-NAFL, MG-NASH, and MG-fibrosis mice, the GXZY-NAFL, GXZY-NASH, and GXZY-fibrosis mice and the nonalcoholic fatty liver fibrosis stage had slightly lighter and narrower bands and reduced protein expression. As represented by the histogram in Figure 9, the TRPM4 expression of the MG-NAFL, MG-NASH, and MG-fibrosis mice in all the three stages of NAFLD was abnormally increased. Compared with that in the MG-NAFL, MG-NASH, and MG-fibrosis mice, the expression level of the TRPM4 protein in the GXZY-NAFL, GXZY-NASH, and GXZY-fibrosis mice was decreased, indicating that the Gexia Zhuyu decoction had a good inhibitory effect on the abnormal expression of TRPM4 at all three stages of NAFLD.

### 3.6. RT-PCR

**Figure 10:**
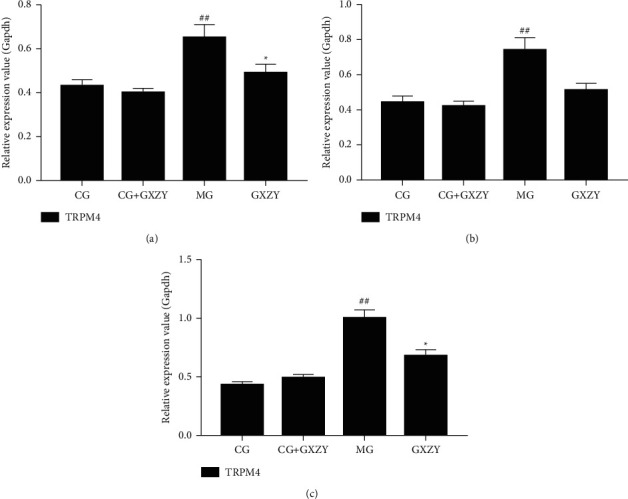
Comparison of the TRPM4 mRNA expression in the liver at the three stages of NAFLD. (a) NAFL group. (b) NASH group. (c) Nonalcoholic fatty liver fibrosis. Compared with the control group, ^##^*P* < 0.01; compared with the model group, ^∗^*P* < 0.05.

Figure 10 shows that the TRPM4 mRNA expression of the MG-NAFL, MG-NASH, and MG-fibrosis mice in all three stages of NAFLD was significantly increased, consistent with the TRPM4 protein expression results from WB. Compared with that of the CG-NAFL, CG-NASH, and CG-fibrosis mice, there was no significant difference in the TRPM4 mRNA expression of the CG-NAFL + GXZY, CG-NASH + GXZY, and CG-fibrosis + GXZY mice. Furthermore, compared with that of the MG-NAFL, MG-NASH, and MG-fibrosis mice, the TRPM4 mRNA expression of the GXZY-NAFL, GXZY-NASH, and GXZY-fibrosis mice was decreased. After drug intervention, the abnormal expression of the TRPM4 mRNA at all three pathological stages of NAFLD was inhibited, with statistically significant differences.

### 3.7. miRNA-24 Regulates TRPM4 Protein Pathway to Improve NAFLD Pathological Stages

#### 3.7.1. Bioinformatic Analysis of TRPM4 Regulation by miRNA-24

**Figure 11:**
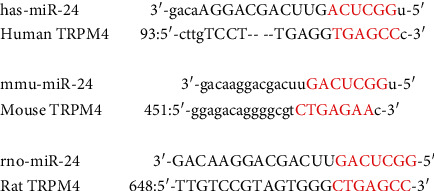
The binding sites of miRNA-24 and the TRPM4 protein.

The strength of miRNA intervention in nonalcoholic fatty liver fibrosis depends on the participation of its target protein in the pathogenesis and development of the disease. Using the database for bioinformatics analysis, it was determined that miRNA-24 and TRPM4 have good binding properties. The specific binding sites are shown in Figure 11.

#### 3.7.2. Detection of miRNA-24 Expression in Liver Tissue by RT-PCR

**Figure 12:**
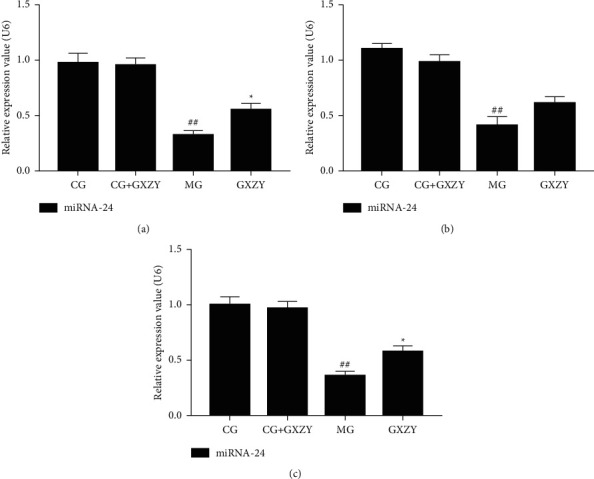
Comparison of liver miRNA-24 content expression at the three stages of NAFLD. (a) NAFL group. (b) NASH group. (c) Nonalcoholic fatty liver fibrosis. Compared with the control group, ^##^*P* < 0.01; compared with the model group, ^∗^*P* < 0.05.

Figure 12 shows that, compared with that of the CG-NAFL, CG-NASH, and CG-fibrosis mice at the three stages of NAFLD, the miRNA-24 expression in the liver tissue of CG-NAFL + GXZY, CG-NASH + GXZY, and CG-fibrosis + GXZY mice did not change significantly. This suggested that the components of the Gexia Zhuyu decoction had no effect on miRNA-24 expression. Compared with that of CG-NAFL, CG-NASH, and CG-fibrosis mice, the miRNA expression of the MG-NAFL, MG-NASH, and MG-fibrosis mice was significantly lower. Furthermore, compared with that of MG-NAFL, MG-NASH, and MG-fibrosis mice, the miRNA-24 expression of the GXZY-NAFL, GXZY-NASH, and GXZY-fibrosis groups was recovered and showed an upward trend. Combining these results with those obtained from the RT-PCR and WB analyses of TRPM4 expression at the three pathological stages of NAFLD, it can be suggested that miRNA-24 expression was normalized after treatment with the Gexia Zhuyu decoction, which can correct the abnormal increase in the TRPM4 channel protein level and reduce the expression of TRPM4 mRNA. These results proved that miRNA-24 regulated the TRPM4 protein pathway.

The experimental results also showed that administration of the Gexia Zhuyu decoction can alter the miRNA-24 expression level at the molecular level, improve the abnormal expression of miRNA-24 in the environment of metabolic disorders, and inhibit the expression of TRPM4 mRNA by upregulating miRNA-24 to maintain the normal cell physiological structure. Thus, these results highlighted the potential role of the Gexia Zhuyu decoction in the treatment of NAFLD.

## 4. Discussion

This study showed that the Gexia Zhuyu decoction, a classic compound used to promote blood circulation and alleviate blood stasis, has a clear therapeutic effect at each pathological stage of NAFLD progression in HFD-fed and MCD-fed mice. The therapeutic mechanism of the Gexia Zhuyu decoction may be related to the targeted regulation of TRPM4 by miRNA-24. Our results suggest that TRPM4 is involved in the complex physiological and pathological mechanisms of NAFLD at various stages. Bioinformatics analysis further revealed that miRNA-24 could target and regulate the TRPM4 pathway, interfere with TRPM4 mRNA expression, and successfully disrupt NAFLD progression.

TG and serum AST and ALT levels can reflect liver damage status in various models [34]. In this study, through diet-induced modeling, serum AST and ALT levels in the MG-NAFL, MG-NASH, and MG-fibrosis mice were found to be increased at all three pathological stages. After Gexia Zhuyu decoction intervention, the ALT, AST, and liver and serum TG levels in CG-NAFL, CG-NASH, CG-fibrosis, and CG-NAFL + GXZY, CG-NASH + GXZY and CG-fibrosis + GXZY mice did not show significant changes, suggesting that the Gexia Zhuyu decoction has almost no toxic effect on the body. The general condition of the GXZY-NAFL, GXZY-NASH, and GXZY-fibrosis mice at each pathological stage improved, compared with that of the MG-NAFL, MG-NASH, and MG-fibrosis mice, and the AST, ALT, and liver TG indicators also decreased. The serum TG levels of the GXZY-fibrosis mice increased, and they showed reduced liver fat deposits. These results indicate that administration of the Gexia Zhuyu decoction can delay continuous liver structure damage and help restore liver physiological functioning.

Lipid formation in blood is positively correlated with serum TC, TG, and LDL levels and negatively correlated with HDL levels [35, 36]. In this study, serum TC and LDL levels decreased, HDL levels increased, and lipid disorders were corrected with drug intervention. The serum TNF-*α* and IL-6 levels in the NAFLD group decreased after drug intervention [37], while in the GXZY-NASH and GXZY-fibrosis groups, SOD levels increased, MDA expression decreased, and the oxidative stress response was inhibited. The PCIII, IV-C, LN, and HA levels reflect not only the degree of liver damage but also the degree of liver fibrosis [38]. After drug intervention, the expression of the above indicators decreased, and the abnormal proliferation of fibrous tissue was inhibited. Pathological sections showed that fatty vacuoles were slightly reduced, and the structures of some liver cells tended to improve.

TRPM4 is widely present in tissues and organs and participates in many complex physiological and pathological processes [39]. Studies have found that TRPM4 protein and mRNA expressions in cells increase when the vascular endothelium is damaged [40, 41], leading to an uninterrupted flow of extracellular Na^+^ into the cell. This, in turn, leads to an increase in the intracellular osmotic pressure and a large influx of H_2_O, causing the cell to swell or rupture [42, 43]. Thus, the TRPM4 protein was highly expressed at all stages of NAFLD, suggesting that it plays an important role in the pathogenesis and development of NAFLD. These findings may guide further studies on chronic metabolic diseases represented by TRPM4 and NAFLD.

The Western blotting results of the liver tissue proteins showed that TRPM4 protein expression levels of MG-NAFL, MG-NASH, and MG-fibrosis mice were significantly increased, indicating that liver damage persisted in the mice. After drug intervention, the expression level of TRPM4 protein significantly reduced, indicating that the Gexia Zhuyu decoction administration regulated TRPM4 protein expression and reduced blood vessel damage, thereby reducing liver tissue damage. Furthermore, the TRPM4 mRNA expression levels of the MG-NAFL, MG-NASH and MG-fibrosis mice were higher than those of the CG-NAFL, CG-NASH and CG-fibrosis mice, and this expression decreased after Gexia Zhuyu decoction intervention, indicating that the TRMP4 channel was activated under pathological conditions, such that TRPM4 mRNA was highly expressed in normal cells. Disruption of normal cell function promotes disease development. After Gexia Zhuyu decoction intervention, both the protein and mRNA levels of TRPM4 reduced. This suggests that treatment with the Gexia Zhuyu decoction regulates the expression of TRPM4 protein and can affect the TRPM4 mRNA expression. Furthermore, the inhibition of TRPM4 protein expression likely maintains the normal physiological structure of the cell.

The abnormal expression of miRNAs, which are important posttranscriptional regulators, is closely associated with chronic liver disease development [44]. Alisi et al. [45] found that the oxidative stress response, inflammatory response, and cell necrosis in an HFD-induced NAFLD models were all related to the abnormal expressions of miRNAs. miRNA-24, a small RNA that is highly conserved in mammals, can reduce actin expression in stress fibers and affect cell migration. miRNA-24 is abundantly expressed in cardiomyocytes and endothelial cells and is involved in heart tissue growth, remodeling, fibrosis, and blood vessel growth and contraction [46].

miRNA-24 expression level in body fluid environments similar to those in NAFLD, such as high salt, high sugar, and high fat, changes with tissue pathological structure. Its expression in the livers of HFD-induced obese mice is significantly upregulated and is involved in the regulation of the cellular lipid metabolism and inflammation [47, 48]. Interestingly, miRNA-24 expression is reduced in endothelial cell dysfunction [49]. In an acute myocardial infarction mouse model, miRNA-24 expression was downregulated in diseased tissues; however, in vivo injection of miRNA-24 could inhibit apoptosis, indicating that the increase in miRNA-24 expression could alleviate myocardial infarction. Therefore, miRNA-24 may be an antiapoptotic miRNA [50]. This experiment proved that miRNA-24 expression increased after Gexia Zhuyu decoction intervention and that the expression of circulating miRNA-24 was adjusted to inhibit the activation of cells at various stages of disease progression.

Through bioinformatic analysis, the target regulation relationship of miRNA-24 on the TRPM4 protein was clarified. The results showed that administration of Gexia Zhuyu decoction restored the mRNA expression of miRNA-24 at all three stages of NAFLD and decreased the protein and mRNA levels of TRPM4, that is, the Gexia Zhuyu decoction intervention could correct the metabolic disorder associated with the progression of NAFLD, recall the expression of circulating miRNA-24 when the expression of miRNA-24 increased, target and regulate the TRPM4 mRNA expression, and inhibit the activation of the TRPM4 pathway in pathological conditions, thereby maintaining the physiological functioning of cells at the various stages of NAFLD progression.

This study has several limitations. First, this study was conducted over a long period of time, mainly because of the tedious process of experimentation, which resulted in the failure of advancement to the next steps. Thus, we could only study the mechanism of one phase of the pathophysiology, i.e., the nonalcoholic fatty liver fibrotic stage. Therefore, in the future, the regulatory mechanisms operating between nonalcoholic fatty liver disease (NAFL) and nonalcoholic steatohepatitis (NASH) and TRPM4 should be comprehensively explored to gain a complete understanding of NAFLD pathophysiology.

## 5. Conclusions

Gexia Zhuyu decoction has obvious therapeutic effects on all stages of NAFLD. The treatment of NAFLD can be achieved by improving lipid metabolism disorders, correcting serological disorders, reducing oxidative stress damage, and decreasing abnormal fibrous tissue proliferation. The TRPM4 channel mainly controls the influx of cations in the cell, regulates the calcium signal of the cell, and thereby participates in the process of cell proliferation and migration. Under the abnormal body fluid environment caused by NAFLD, there are large amounts of extracellular Ca^2+^ influxes, and TRPM channel activation leads to endothelial cell dysfunction and related inflammatory reactions. As shown by studies, inhibiting the abnormal elevation of TRPM4 in pancreatic *β*-cells can not only significantly reduce glucose-induced insulin secretion but also alleviate insulin resistance by regulating Ca^2+^ signals.

Gexia Zhuyu decoction can increase the expression level of miRNA-24, target and regulate TRPM4 protein channel, improve the abnormal activation state of TRPM4 channel, silence TRPM4 mRNA expression, promote intracellular Na^+^ and Ca^2+^ concentration and membrane potential changes to return to normal, reduce cells' internal ROS levels, improve liver cell damage caused by NAFLD at various stages, and correct metabolic dysfunction.

## Figures and Tables

**Table 1:** Primer sequences.

Gene	Forward primer	Reverse primer
*Trpm*4	5′-GACTGCCTTCCTGGGTAATG-3′	5′-CTCGGAGACACTTGGCTTTG-3′
*Actb*	5′-GGCACCACACCTTCTAC-3′	5′-CTGGGTCATCTTTTCAC-3′
*Gapdh*	5′-ATCACTGCCACCCAGAAG-3′	5′-TCCACGACGGACACATTG-3′
*Mir*24-1	5′-TGGCTCAGTTCAGCAGGAACAG-3′	5′-GATCCAGTCTCAGGGTCCGAG-3′
*RNU*6-1	5′-CTCGCTTCGGCAGCACA-3′	5′-AACGCTTCACGAATTTGCGT-3′

**Table 2:** Serum biochemical indicators after Gexia Zhuyu decoction administration.

nmol/L	NAFL				NASH				Nonalcoholic fatty liver fibrosis			
	CG	CG + GXZY	MG	GXZY	CG	CG + GXZY	MG	GXZY	CG	CG + GXZY	MG	GXZY
Serum ALT	50.0 ± 0.05	48.32 ± 1.13	122.3 ± 8.35	95.26 ± 6.03^*∗*^	37.35 ± 6.16	36.68 ± 5.58	235.57 ± 7.32	202.11 ± 7.39^*∗∗*^	39.32 ± 1.45	42.05 ± 1.62	224.51 ± 18.16	127.53 ± 10.12^*∗∗*^
Serum AST	105.55 ± 5.30	103.26 ± 4.83	196.28 ± 3.47	165.52 ± 5.02^*∗∗*^	105.63 ± 6.49	108.05 ± 6.37	298.51 ± 11.16	254.53 ± 10.12^*∗∗*^	86.35 ± 9.16	84.68 ± 5.58	285.57 ± 15.32	152.11 ± 7.39^*∗∗*^
Serum TG	0.73 ± 0.12	0.75 ± 0.09	1.75 ± 0.08	1.23 ± 0.16^*∗∗*^	0.91 ± 0.21	0.89 ± 0.22	1.97 ± 0.13	1.45 ± 0.14^*∗∗*^	0.95 ± 0.21	0.91 ± 0.24	0.57 ± 0.17	0.78 ± 0.25^*∗*^
Liver TG	0.85 ± 0.12	0.82 ± 0.09	1.65 ± 0.29	1.12 ± 0.06^*∗*^	0.85 ± 0.12	0.82 ± 0.09	2.05 ± 0.08	1.69 ± 0.16^*∗∗*^	3.23 ± 0.30	3.57 ± 0.32	7.59 ± 0.34	5.41 ± 0.25^*∗*^

Compared to the model group, ^∗∗^*P* < 0.01, ^∗^*P* < 0.05.

## Data Availability

The datasets used and/or analyzed during the current study are available from the corresponding author upon request.
